# Lung-Derived Microscaffolds Facilitate Diabetes Reversal after Mouse and Human Intraperitoneal Islet Transplantation

**DOI:** 10.1371/journal.pone.0156053

**Published:** 2016-05-26

**Authors:** Nasser Abualhassan, Lena Sapozhnikov, Rena L. Pawlick, Meygal Kahana, Andrew R. Pepper, Antonio Bruni, Boris Gala-Lopez, Tatsuya Kin, Eduardo Mitrani, A. M. James Shapiro

**Affiliations:** 1 Alberta Diabetes Institute, University of Alberta, Edmonton, AB, Canada; 2 Department of Cell and Developmental Biology, The Hebrew University of Jerusalem, Jerusalem, Israel; 3 Clinical Islet Transplant Program, University of Alberta, Edmonton, AB, Canada; Children's Hospital Boston/Harvard Medical School, UNITED STATES

## Abstract

There is a need to develop three-dimensional structures that mimic the natural islet tissue microenvironment. Endocrine micro-pancreata (EMPs) made up of acellular organ-derived micro-scaffolds seeded with human islets have been shown to express high levels of key beta-cell specific genes and secrete quantities of insulin per cell similar to freshly isolated human islets in a glucose-regulated manner for more than three months *in vitro*. The aim of this study was to investigate the capacity of EMPs to restore euglycemia *in vivo* after transplantation of mouse or human islets in chemically diabetic mice. We proposed that the organ-derived EMPs would restore the extracellular components of the islet microenvironment, generating favorable conditions for islet function and survival. EMPs seeded with 500 mouse islets were implanted intraperitoneally into streptozotocin-induced diabetic mice and reverted diabetes in 67% of mice compared to 13% of controls (p = 0.018, n = 9 per group). Histological analysis of the explanted grafts 60 days post-transplantation stained positive for insulin and exhibited increased vascular density in a collagen-rich background. EMPs were also seeded with human islets and transplanted into the peritoneal cavity of immune-deficient diabetic mice at 250 islet equivalents (IEQ), 500 IEQ and 1000 IEQ. Escalating islet dose increased rates of normoglycemia (50% of the 500 IEQ group and 75% of the 1000 IEQ group, n = 3 per group). Human c-peptide levels were detected 90 days post-transplantation in a dose-response relationship. Herein, we report reversal of diabetes in mice by intraperitoneal transplantation of human islet seeded on EMPs with a human islet dose as low as 500 IEQ.

## Introduction

Type 1 diabetes mellitus (T1DM) is a chronic autoimmune disorder characterized by destruction of pancreatic β-cells and insulin deficiency.[1, 2] Life-long exogenous insulin replacement remains standard management. While intensive insulin treatment delays microvascular complications, it significantly increases risk of severe hypoglycemic events that can be disabling and occasionally fatal.[3–5]

In the past decade, pancreatic islet transplantation has shown promising outcomes with 5-year insulin independence rates approaching 50% in selected centers.[6, 7] To date, intrahepatic islet infusion via the portal vein is the only clinically approved site that has routinely resulted in insulin independence.[7, 8] In spite of recent advancements in islet transplantation, up to 70% of transplanted islets fail to engraft within the early post-transplant period.[5, 9] A major contributor to initial loss is the innate instant blood-mediated inflammatory reaction (IBMIR), resulting from exposure of islets to blood.[10, 11] Identifying alternative sites for islet transplantation could potentially ameliorate this effect, thereby reducing islet loss. Furthermore, the process for islet isolation and purification disrupts islet vasculature and injures the local microenvironment, further compromising engraftment.[12, 13]

The islet microenvironment is composed of a peri-insular basement membrane (BM) and extracellular matrix (ECM).[14–16] The ECM is a complex of different molecules that serves as a physical site for attachment and support, as well as a framework for cellular proliferation, differentiation and communication.[14, 17–20] The ECM binds and stores many cytokines, growth factors and other signaling molecules that modulate cellular behavior.[16, 21] Loss of peri-insular BM and apoptosis are evident immediately after enzymatic islet digestion.[17–19] Collagen-IV, laminin and fibronectin are the most commonly reported components of this microenvironment.[16, 17] Multiple studies have shown enhanced *in vitro* islet function for islets co-cultured with ECM components including collagen-IV, fibronectin, laminin, thrombospondin and heparin sulfate.[22–26] Islets embedded within a collagen gel maintain their spherical structure and secretory capacity compared to islets cultured under standard conditions.[27] As demonstrated by Wang *et al*., the apoptotic index, was significantly higher for islets cultured in standard conditions compared to islets co-cultured with collagen or fibronectin.[17]

Although interaction between islets and their surroundings is complex and incompletely understood, supplementing transplanted islets with ECM components and restoring the three-dimensional (3D) architecture appears to have a beneficial effect as evidenced by improved viability and function. [28–31] Islets seeded on a poly(dimethylsilonxane) 3D scaffold, collagen matrix or fibroblast populated collagen matrix have shown improved *in vivo* function.[28–30] Salvay *et al*. seeded islets on microporous, biodegradable poly(lactide-co-glycolide) (PLGA) scaffolds coated with collagen-IV, fibronectin or laminin and found that diabetic mice exhibited significantly shorter time to restore normoglycemia compared to controls.[31]

Recently, organ-derived microscaffolds have been prepared from decellularized lung tissue, engineered endocrine micro-pancreata (EMPs), and subsequently seeding with human islets was shown to function significantly better than free islets.[32, 33] EMPs have also been found to express high levels of key beta-cell specific genes and secrete quantities of insulin per cell similar to freshly isolated human islets in a glucose-regulated manner for more than three months *in vitro*.[32] Lung instead of pancreas-derived micro-scaffolds were chosen since most of the pancreatic matrix is derived from the exocrine organ [34] and the lung matrix may enable interaction between beta cells and endothelial cells.[35]

In this study, we sought to evaluate the function of the EMPs after implantation into hyperglycemic mice. We hypothesized that the EMPs would provide essential ECM macromolecules and structural support to maintain islet viability *in vivo*. We explored this approach with both mouse and human islets transplanted into immunodeficient mice.

## Materials and Methods

### Mouse Islet Isolation

All animals were housed under conventional conditions having free access to food and water. The care of the mice was in accordance with the guidelines approved by the Canadian Council on Animal Care. All experimental procedures were approved by the University of Alberta Research Ethics and Animal Use Committee (Study ID: AUP00000419). Pancreatic islets were isolated from 8 to 12 week male BALB/c mice (Jackson Laboratories, CA). Before pancreatectomy, the common bile duct was cannulated and the pancreas was distended with 0.125 mg/mL cold Liberase TL Research Grade enzyme (Roche Diagnostics, Laval, QC, CA) in Hanks balanced salt solution (Sigma, St. Louis, MO, USA). Islets were isolated by digesting the pancreata at 37°C for 14 minutes with light shaking. Subsequent to the digestion phase, islets were purified from the pancreatic digests using histopaque-density gradients (1.108, 1.083 and 1.069 g/mL, Sigma, St. Louis, MO, USA). Islets were cultured in CMRL-1066 (Corning-cellgro, Manassas, VA, USA) supplemented with 10% fetal bovine serum, 1% L-glutamine (200 mM/L, Sigma, St. Louis, MO, USA), 1% sodium pyruvate (100 mM, Sigma, St. Louis, MO, USA), 1% non-essential amino acid 100x (Sigma, St. Louis, MO, USA), 100 U/mL penicillin-G and100 μg/mL streptomycin (Sigma Aldrich Canada Co., Oakville, ON, CA). A total of 5 mouse islet isolations were performed and all groups were randomized to receive islets from each isolation.

### Human Islet Isolation

Human islets were isolated from a human pancreas procured from a multi-organ deceased donor transported to the clinical isolation center in cold preservation solution. The human islets were isolated implementing a modified Ricordi technique.[36, 37] All work was approved through the Health Research Ethics Board—Biomedical Panel of the University of Alberta. Written permission was obtained in all cases from the organ donor’s family to use islets for experimental research. Processed human islets were only made available for research after failing to yield minimal mass required for clinical transplantation. Human islets were cultured in clinical grade CMRL-1066 media (Media Tech, MT99-603-L) supplemented with insulin selenium-transferrin and insulin-like growth factor-1 at 22°C and were received 24 hours after isolation.

### Preparation of Decellularized Microscaffolds

Both human cadaveric and porcine lung derived 3D microscaffolds were engineered and prepared according to previously established protocols.[32, 33] Briefly, human and porcine lung tissue were stored at -80°C until required. The frozen tissue was cut into 5–8 mm diameter cylinders with a core drill press. The cores were cut into small slices, approximately 300 μm in thickness and decellularized according to previously described methods.[32] The microscaffolds were stored at 4°C overnight in PBS supplemented with 1000 U/mL penicillin-G, 1 mg/ml streptomycin (Sigma Aldrich, St. Louis USA). Mouse islets were seeded on porcine decellularized lung tissue, while human islets were seeded on human decellularized lung tissue derived from discarded surgical resection or cadaveric-derived lungs that could not be used for clinical transplantation. Permission for use of human cadaveric lung tissue was obtained through the Human Health Research Ethics Board—Biomedical Panel of the University of Alberta (Study ID: Pro00041552). Written permission was obtained in all cases from the organ donor’s family to use the lung for experimental research. The lung was made available for research only after it was deemed unsuitable for clinical transplantation.

### Seeding of Islets onto Microscaffolds

Prior to seeding, decellularized microscaffolds were washed three times with PBS to remove excess antibiotics then cultured in medium described above at 37°C, 5% CO_2_ and saturated humidity for a minimum of 2 hours. For the pilot study, islets were seeded manually by pipetting the required islet dose directly onto the scaffold in minimal culture medium. For the remainder of the study, islets were aliquoted and re-suspended in low volume (3 ml) culture medium. The aliquoted islets and microscaffolds were transferred to a 500 cc glass pyrex bottle targeting 50 islets per micro-scaffold. The bottle was rotated at 5 rotations/minute on a roller-mixer (SRT9D, Stuart) at 37°C, 5% CO_2_ and saturated humidity for 90 minutes. The seeded microscaffolds referred to as endocrine micro-pancreata (EMPs).

### Transplantation with Mouse Islets

Diabetes was chemically induced with streptozotocin (175 mg/kg i.p.) (Sigma, St. Louis, MO, USA) in adult immunodeficient C57BL/6 RAG^-/-^ mice (Jackson Laboratories, Bar Harbor, ME, USA) at 12–14 weeks of age. Animals were considered diabetic after two consecutive non-fasting blood glucose measurements >15 mmol/L.

All transplant recipients received 500 islets ± 10% with a purity of 90% and were divided into three groups: a positive control group (n = 9) with islet transplantation under the kidney capsule (KC500), a control group (n = 8) with free islet transplantation into the intraperitoneal (IP500) cavity on the liver and stomach surfaces and a study group (n = 9) with EMP implantation into the peritoneal cavity (EMP500). A fraction of the EMPs (10%) were removed from the study, stained with dithizone and enumerated to extrapolate the number of EMPs per transplant (3 mg/ml dithizone, Sigma Aldrich Canada Co., Oakville, ON, CA).

### Transplantation with Human Islets

A pilot study was initially conducted at the Hebrew University of Jerusalem by Dr. Eduardo Mitrani to explore the potential impact of EMPs on islet function *in vivo*. 12 NOD-SCID mice (Charles River, Hollister, CA, USA) were implanted with EMPs subcutaneously at three doses: 150 islet equivalents (IEQ) ± 10%, 200 IEQ ± 10%, 500 IEQ ± 10% and control empty scaffolds (n = 3 per group). Human islets were manually seeded onto the EMPs for the pilot study. The second cohort of experiments was conducted at the University of Alberta using adult immunodeficient C57BL/6 RAG^-/-^ mice (Jackson Laboratories, Bar Harbor, ME, USA). Diabetes was chemically induced with streptozotocin (175 mg/kg i.p.) (Sigma, St. Louis, MO, USA) for all mice and animals were considered diabetic after two consecutive non-fasting blood glucose measurements >15 mmol/L. Mice were separated into 3 groups: low dose Group A received EMPs seeded with a marginal therapeutic dose of 250 IEQ ± 10% (n = 4), intermediate dose Group B received EMPs seeded with 500 IEQ ± 10% (n = 4) and high dose Group C received EMPs seeded with 1000 IEQ ± 10% (n = 4). Outcomes were compared with concurrent and recent historic controls from our laboratory of similar strain mice receiving 1000 IEQ ± 10% (n = 17) human islets transplanted beneath the renal capsule. All EMPs were implanted within the peritoneal cavity on the liver surface.

### Assessment of Graft Function

After transplantation, non-fasting blood glucose levels were monitored three times per week (between 13:00 and 17:00) using a portable glucometer (OneTouch Ultra 2, LifeScan, CA, USA). Mice were considered normoglycemic at blood glucose levels maintained at <11.1 mmol/L throughout the study period. Intraperitoneal glucose tolerance tests (IPGTTs) were performed 6 weeks post-transplant to assess the capacity of the graft to respond to a glucose bolus. After 8 hours of fasting, mice were injected with 3 g/kg 25% dextrose intraperitoneally. Blood glucose levels were monitored at 0, 15, 30, 60 and 120 minutes post-dextrose infusion. A recovery nephrectomy was performed on all mice that received transplantation under the kidney capsule to confirm graft-dependent function. For mice transplanted with human islets, blood samples were obtained by cardiac puncture at the time of euthanasia (90 days post-transplantation) and human c-peptide levels were measured by ELISA (Ultrasensitive, Mercodia, Uppsala, Sweden). Normoglycemic mice received 3 g/kg 25% dextrose 15 minutes prior to collection.

### Relative Quantitative Real-Time Polymerase Chain Reaction

RT-PCR was performed as previously described by *Sionov et al* [32]. RNA was isolated by TriReagent (Sigma, St. Louis, MO, USA) then converted to cDNA using AB high capacity kit (Applied Biosystems). High precision TaqMan primers and TaqMan Gene Expression Master Mix in an ABI PRISM 7900HT Sequence Detection System (Applied Biosystems) were used (Table 1). Since no significant differences were observed between the three different housekeeping genes, TATA-box binding protein (TBP), GAPDH and HPRT used as internal standards, TBP was used.

**Table 1:** Reference sequences of TaqMan probes obtained from the Applied Biosystems TaqMan expression system.

Gene	Accession number	ABI primer RefSeq
TBP	**NM_003194.4**	**Hs99999910_m1**
GAPDH	**NM_002046.4**	**Hs99999905_m1**
HPRT-1	**NM_000194.2**	**Hs02800695_m1**
Insulin	**NM_000207.2**	**Hs02741908_m1**
Pdx-1	**NM_000209.3**	**Hs00236830_m1**

The threshold Cycle (Ct) of each gene for a given EMP sample was subtracted from the Ct of TBP of the same sample (ΔCt), which was then subtracted from the ΔCt of the donor islet sample (ΔΔCt). The fold change in gene expression was calculated by the power of 2 of the–ΔΔCt value (2^-ΔΔCt^). Thus, the presence of other cell types within the EMP leads to a reduction in the insulin/TBP ratio.

### Histological Analysis

At 60 days post-transplantation, EMPs containing mouse islet grafts were explanted, fixed in formalin and subsequently embedded in paraffin. 5 μm sections were prepared and stained with Masson’s trichrome to visualize connective tissue. Immunofluorescent double staining was performed using primary antibody of guinea pig anti-pig insulin (1:100; Dako, Carpinteria, CA, USA) and rabbit anti- CD31 (1:50; abcam, Cambridge, MA, USA) overnight at 4°C. Secondary antibody of goat anti-guinea pig (1:200; Rhodamine, Jackson ImmunoResearch Laboratories) and goat anti-rabbit (1:200; Fluorescein, Vector Laboratories, Burlingame, CA, USA) were used on the second day of staining. Samples were counterstained with DAPI in anti-fade mounting medium (ProLong, LifeTechnologies, Eugene, OR, USA). Slides were examined under fluorescent microscopy. Images were photographed using the appropriate filter with AxioVision imaging software. For EMPs seeded with human islets, grafts were explanted 90 days post-transplantation and processed as mentioned above. In addition to insulin staining, the grafts were double stained with rabbit anti-glucagon (1:200; abcam, MA, USA) for 2 h at 4°C. Secondary antibody consisting of goat anti-guinea pig (1:200 Rhodamine, Jackson) and goat anti-rabbit (1:200; Fluorescein, Vector Laboratories, Burlingame, CA, USA)

### Statistical Analysis

Data are represented as means ± standard error of the mean (SEM). Area under the curve (AUC) for IPGTTs and differences between groups were calculated using one-way ANOVA with Tukey’s post-hoc test. One-way ANOVA and Tukey’s post-hoc test was used to compare human c-peptide levels as well. Kaplan-Meier survival function curves were compared using the log-rank statistical method. Statistical analyses were performed using GraphPad Prism (GraphPad Software, La Jolla, CA, USA). A p value < 0.05 was considered significant.

## Results

### Subcutaneous implantation of EMPs reversed hyperglycemia in NOD-SCID mice

**Fig 1:**
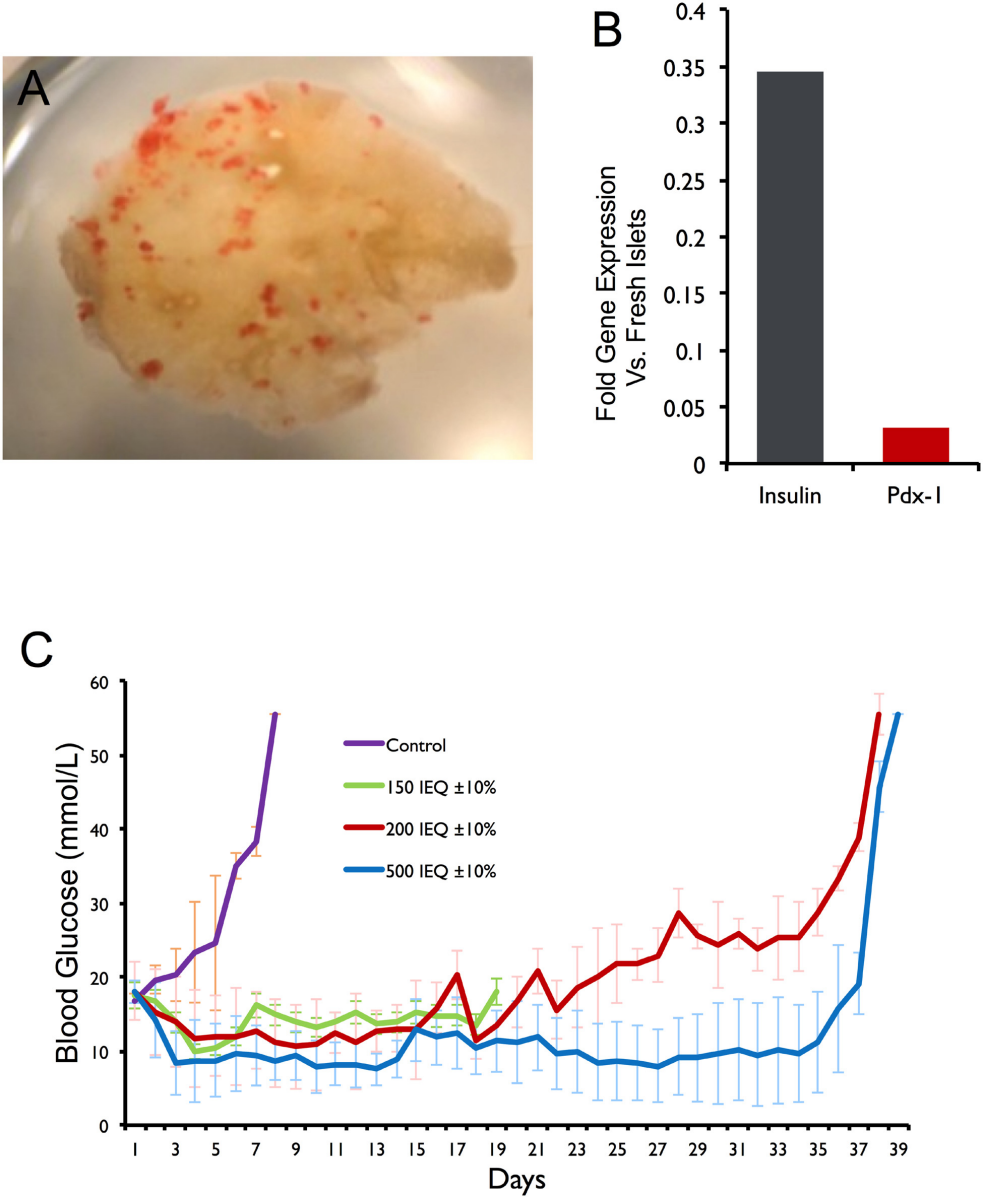
Pilot study of endocrine micro-pancreata (EMPs) implanted subcutaneously. (A) Dithizone stained EMP showing numerous human islets (red) seeded on the microscaffold. (B) Real time PCR gene expression of insulin and PDX-1 normalized to house-keeping gene TBP. Values are presented as fold expression per cell compared the values obtained from fresh islets (n = 3, single analysis of pooled EMPs removed from three test animals). (C) Average non-fasting blood glucose levels for the three EMP doses implanted subcutaneously. Data are presented as mean ± SEM. This part of the study was completed at the Hebrew University of Jerusalem by or collaborators.

A pilot study was conducted at the Hebrew University of Jerusalem by Dr. Eduardo Mitrani to investigate the potential benefits of the EMPs on *in vivo* islet function. Dithizone staining of the EMPs confirmed the presence of islets (Fig 1A). Mice receiving 150 IEQ were followed for 19 days post-transplant, primarily to evaluate the incorporation of EMPs into the host and the function of this novel approach. None of the animals in this group achieved normoglycemia, and were subsequently euthanatized to retrieve the EMPs. On macroscopic examination, the EMPs became vascularized and exhibited no signs of overt inflammation (data not shown). Real time PCR further confirmed the presence of viable human islets and beta-cell-specific function, as evidenced by transcription of insulin and pancreatic and duodenal homeobox 1 (PDX-1)—a transcription factor that plays a critical role in pancreatic development, beta cell maturation, survival, and the expression of beta- specific genes (Fig 1B). The insulin transcripts per cell were about 35% of fresh islets, which is relatively high taking into consideration that upon removal, the EMP implants by necessity contain host cells incorporated during the vascularization process, which dilute the relative number of Insulin and Pdx-1 copies per cell in the explant. Mice receiving 200 IEQ had reduced blood glucose levels in the first week of implantation (Fig 1C). However, mice reverted to hyperglycemia by three weeks post-implant, most likely due to the small amount of transplanted islets. In contrast, mice receiving 500 IEQ were normoglycemic throughout the study period until the EMPs were retrieved 35 days post-transplantation (Fig 1C). In contrast, non-transplanted hyperglycemic control animal blood glucose levels increased rapidly leading to mortality within 5 to 8 days (Fig 1C).

### EMPs Improve Mouse Islet Graft Function After Intraperitoneal Transplantation

**Fig 2:**
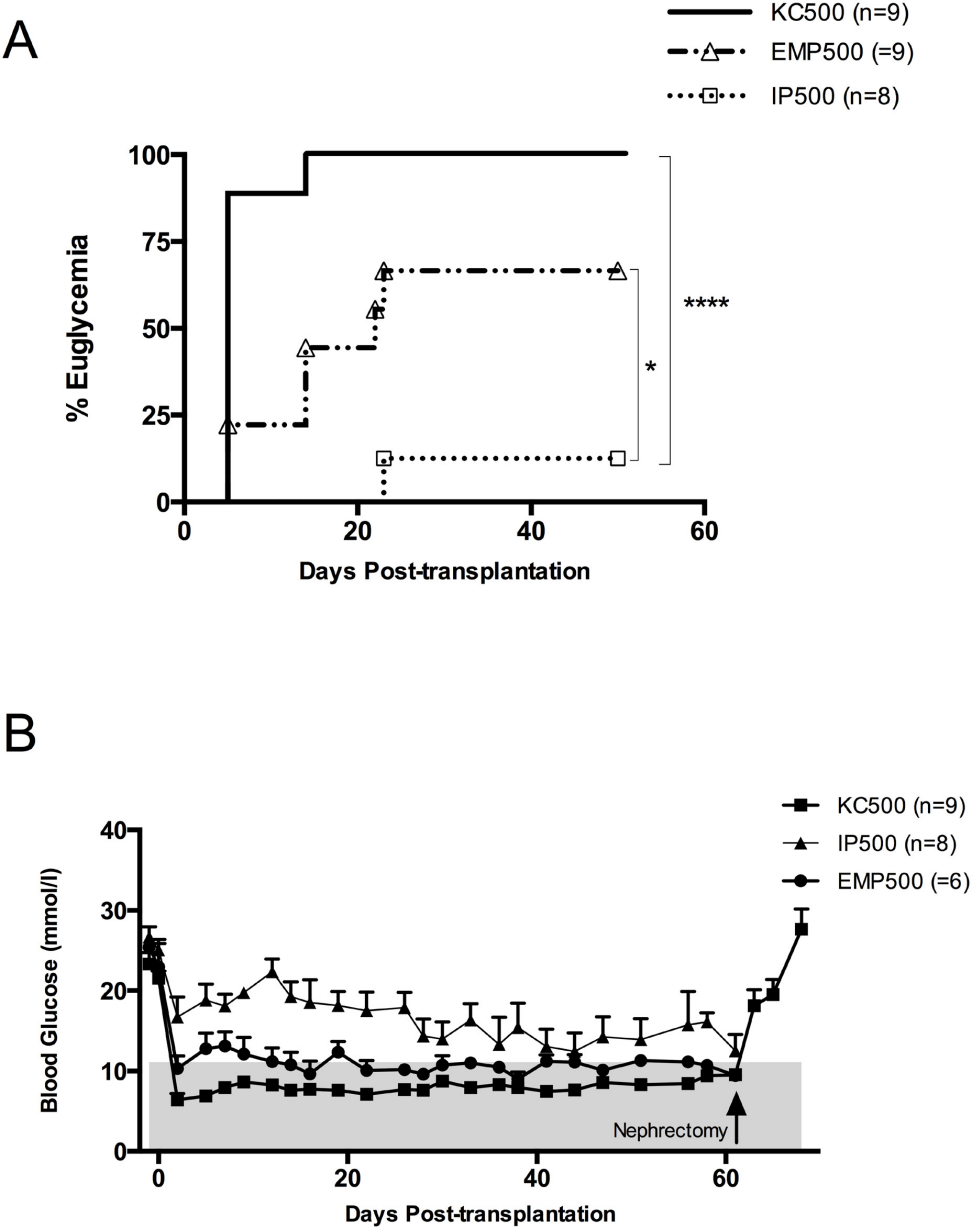
Long-term graft function after mouse islet transplantation. (A) The proportion of animals that achieved normoglycemia. Normoglycemia was restored in 6 animals from the EMP group compared to 1 from the IP group. This difference was statistically significant (P = 0.0183, Log-rank, Mantel-Cox test). All animals from the kidney capsule group were restored to normoglycemia compared to EMP and IP groups (P< 0.01 & 0.0001 respectively, Log-rank, Mantel-Cox test). Transplanted islets were from five different mouse isolations (n = 30 pancreata per isolation). (B) Average non-fasting blood glucose levels for kidney capsule group (KC500), intraperitoneal free islets group (IP500) and seeded microscaffold group (EMP500). Data are presented as mean ± SEM (one-sided error bars for clarity).

An immunodeficient mouse model was used in this study to investigate the impact of EMPs on islet neovascularization and engraftment, without confounding effects from either rejection or immunosuppressive agents. 66.7% of the mice transplanted with EMPs into the peritoneal cavity achieved normoglycemia (n = 9) compared to only 12.5% (n = 8) of the mice receiving free islets IP (p = 0.018) (Fig 2A). All 9 mice receiving murine islets under the kidney capsule became euglycemic. All mice were maintained until 60 days post-transplantation. For islets transplanted under the kidney capsule, used as a positive control, all mice underwent recovery nephrectomies and reverted to hyperglycemia within 48 h (Fig 2B). However, recovery graft retrieval was not technically feasible in the EMP cases due to liver capsular adherence.

### EMPs Improve Mouse Islet Graft Response to Glucose Challenge

**Fig 3:**
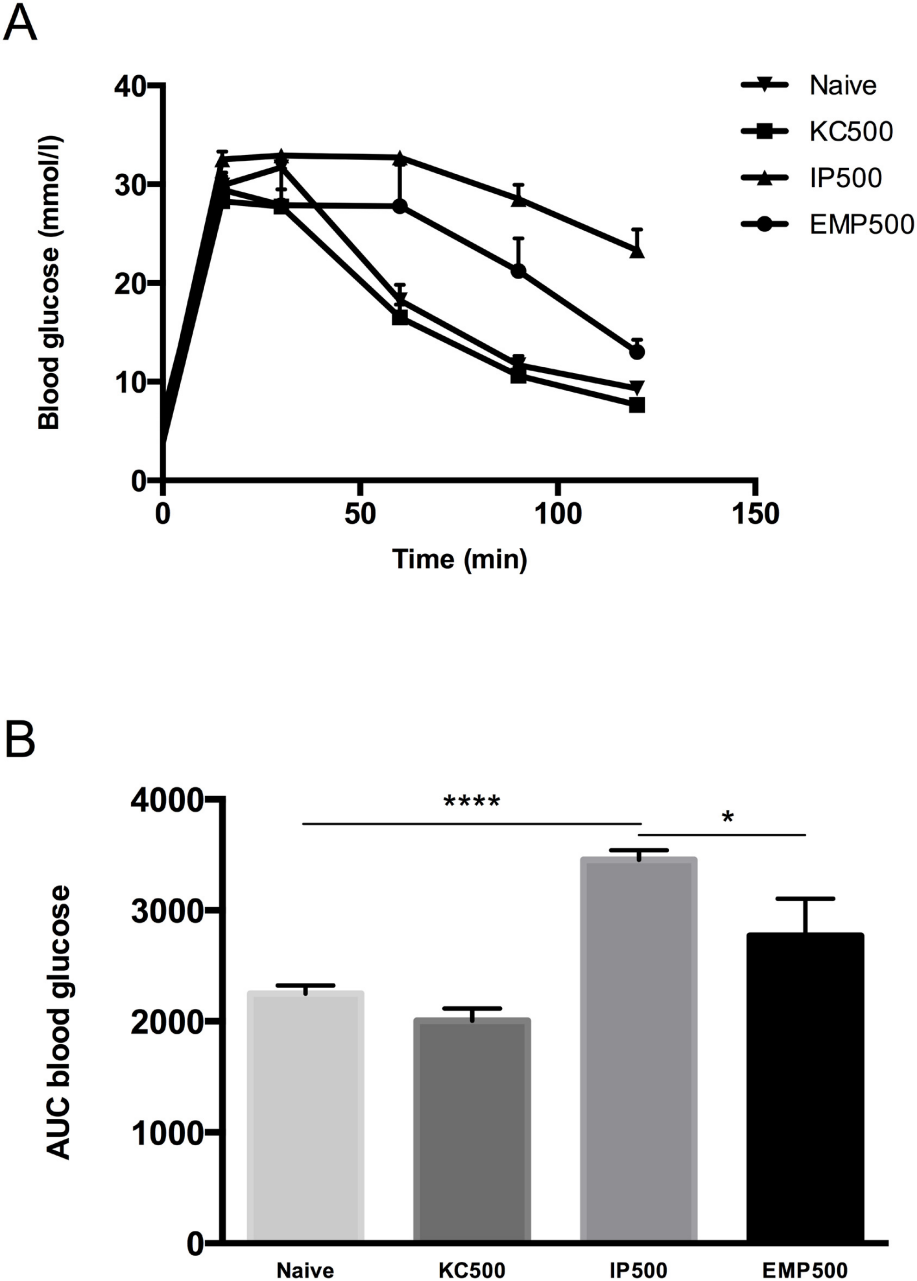
IPGTTs of the transplanted mouse islets six weeks post-transplantation. Blood glucose measurements after dextrose bolus (A) and AUC analysis (B) did not differ between Naïve (n = 5) and EMP (n = 6) groups (p> 0.05, One way ANOVA with Tukey’s post hoc test). Animals that received free intraperitoneal islets (n = 8) were intolerant to glucose challenge compared to EMP, Naïve and KC (n = 9) groups (*p < 0.05, *** p< 0.001, and ****p< 0.0001 respectively; One way ANOVA with Tukey’s post hoc test). All mice received 3 g/kg 25% dextrose i.p. bolus for this test and blood glucose measurements were taken at t = 0, 15, 30, 60, 90 and 120 min. Data are presented as mean ± SEM.

Six weeks post-transplantation, mice underwent IPGTTs to evaluate graft function. IPGTTs were also performed on normoglycemic, age-matched, naïve mice as a control group (n = 5). Blood glucose levels were lower in the EMP transplant group at all time points compared to the free IP islet group (Fig 3A). The area under the curve (AUC) for the EMP group was similar (p> 0.05, One way ANOVA with Tukey’s post hoc test) to that of naïve mice but significantly lower (p < 0.05, One way ANOVA with Tukey’s post hoc test) than the free IP islet transplant group (Fig 3B).

### EMPs Support Islet Architecture

**Fig 4:**
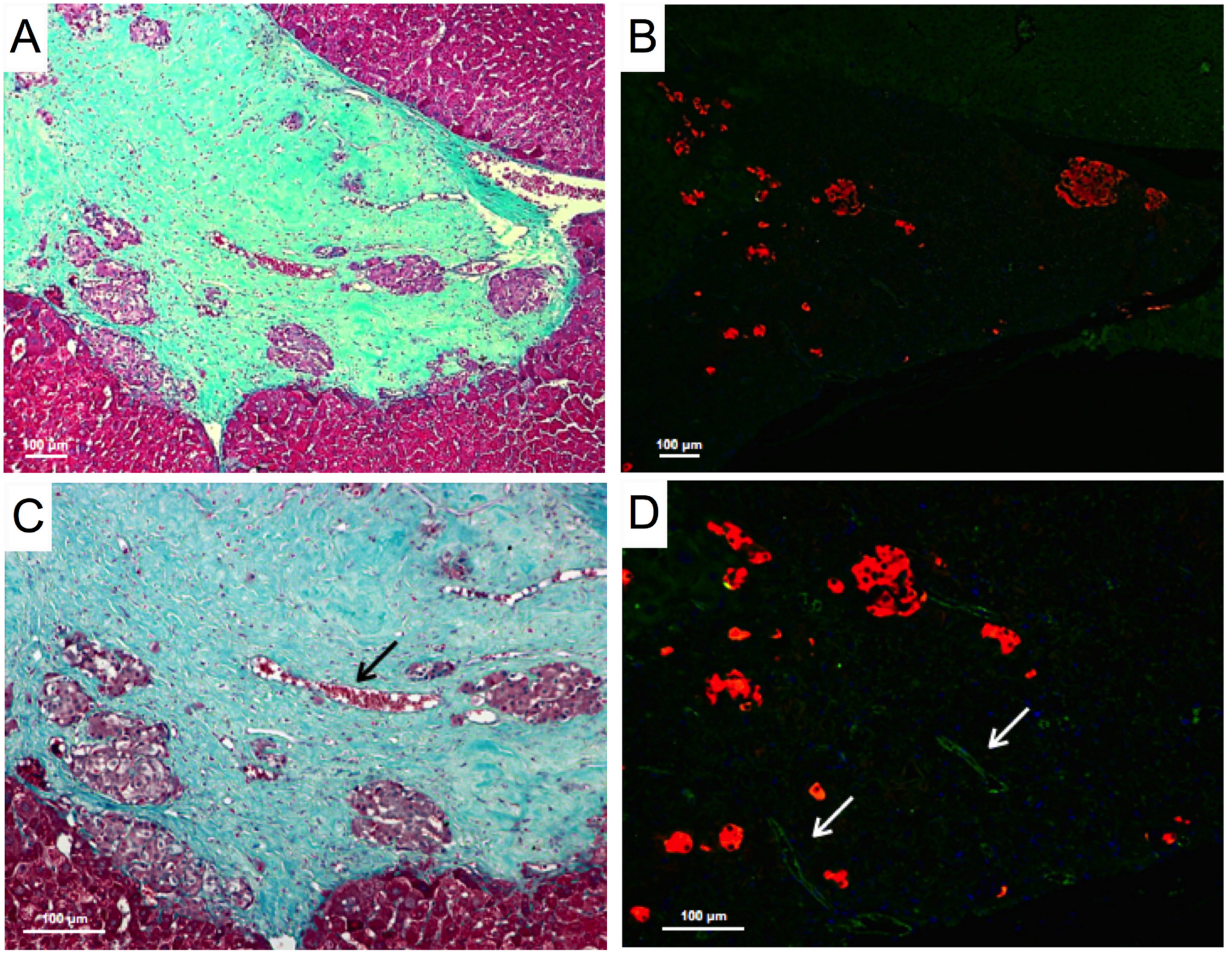
Histological analysis of explanted islet grafts 60 days post-transplantation. (A) Mason’s trichrome staining of a cross-section of explanted EMP showing mouse islets of normal structure and size with surrounding background of collagen (blue), smooth muscles, erythrocytes (red) and scaffold-liver interface at (100x). (C) Mason’s trichrome staining of mouse islets seeded on EMP at higher magnification (200x) showing erythrocyte filled blood vessel (arrow) with fluorescent staining of the same sections (B&D) to confirm the presence of insulin (red) and neovascularization (arrows) with positive anti-CD31 staining.

Islets seeded onto microscaffolds (EMPs) and subsequently transplanted exhibited normal morphology 60 days post-transplantation (Fig 4). An abundance of blood vessels were seen around the islets in the connective tissue-rich background (Fig 4A and 4C). Immunohistochemistry staining revealed a large number of insulin positive islets on the EMPs and stained positive for anti-CD31, which is primarily concentrated at the borders of endothelial cells (Fig 4B and 4D).

### Micro-scaffolds Improve Human Islet Function After Intraperitoneal Transplantation

**Fig 5:**
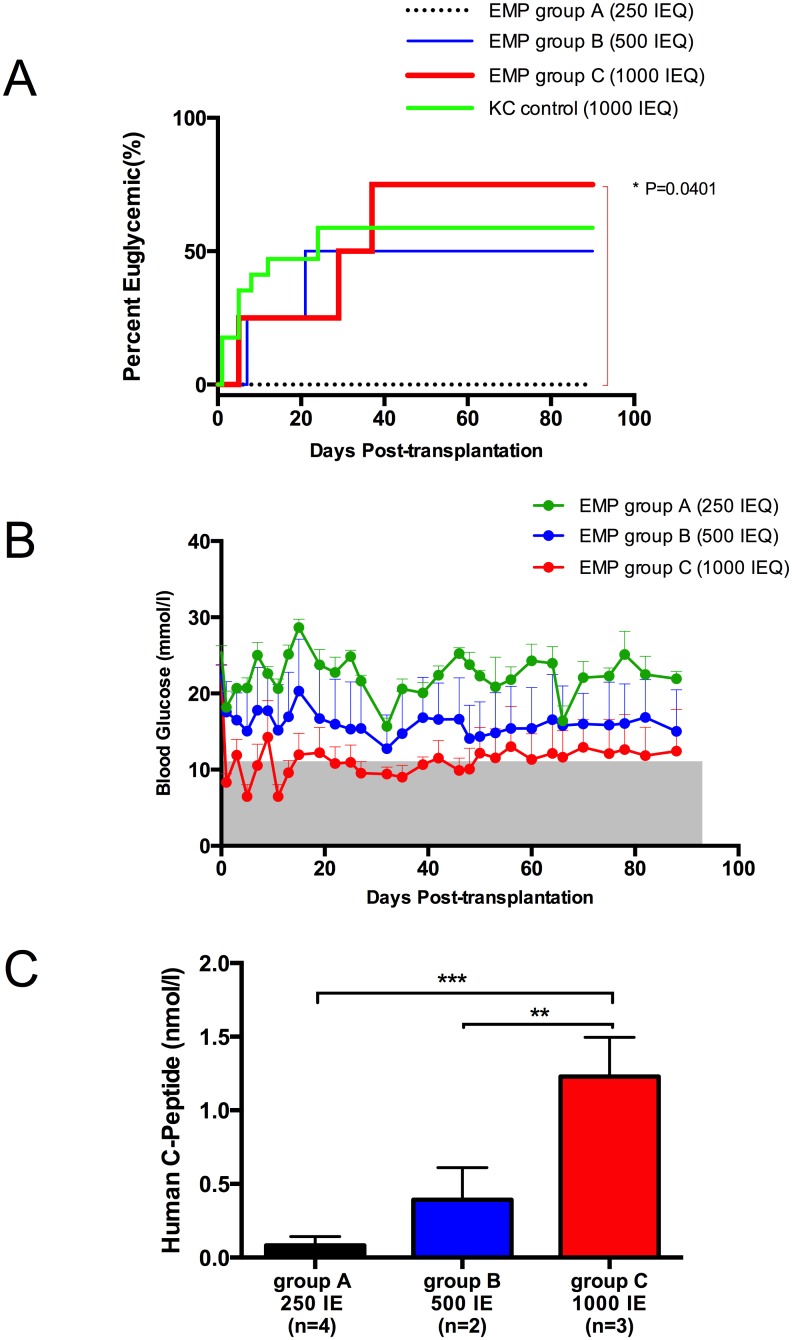
Long-term graft function after human islet transplantation. (A) The proportion of animals that achieved normoglycemia from the high dose EMP group C was significantly higher (p = 0.0401) compared to the low dose EMP group A. However, this difference was not significant compared to the intermediate dose EMP group B and historical kidney capsule groups (p = 0.671 and 0.889; respectively). (B) Average non-fasting blood glucose levels for all the groups were inversely proportional to transplanted islet dose. (C) Average stimulated human C-peptide levels for Group C was significantly higher than Groups A and B (p<0.001 and p<0.01, respectively; one way ANOVA with Tukey’s multiple comparison test).

To further investigate impact of EMPs on intraperitoneal islet engraftment, we sought to determine EMP engraftment using human islets transplanted into immunodeficient mice. The human islet preparation had a purity of 69% and viability of 85%. Mice received low (250 IEQ), moderate (500 IEQ) or high (1000 IEQ) dose EMPs. Three mice receiving high dose EMPs (Group C) achieved normoglycemia (75%, n = 4) as compared to 2 mice receiving moderate dose EMPs (Group B) (50%, n = 4). All mice transplanted with low dose EMPs (Group A) failed to achieve normoglycemia throughout the study (0%, n = 4). Compared to concurrent and recent historic renal subcapsular controls, 10 0f 17 mice (58.8%) achieved normoglycemia with transplantation of a marginal human islet dose of 1000 IEQ (Fig 5A).

As expected, average non-fasting glucose levels were inversely proportional to transplanted islet dose (Fig 5B). To confirm graft-dependent normoglycemia human c-peptide levels were measured in blood samples obtained by cardiac puncture at time of euthanasia 90 days post-transplantation. Seeding EMPs with higher islet mass resulted in higher stimulated human c-peptide levels (Fig 5C). The average stimulated human c-peptide levels for group C was 1.23 ± 0.15 nmol/L compared to 0.39 ± 0.15 nmol/L and 0.08 ± 0.03 nmol/L for group B and A, respectively, confirming a dose-dependent response (Fig 5C). This difference was statistically significant (p<0.01 and p< 0.001, respectively). The observed stimulated human c-peptide levels 90 days post-transplant corresponded to euglycemic function observed through daily non-fasting blood glucose levels (Fig 5B).

### EMPs Maintained Insulin/Glucagon Positive Islets 90 Days Post-Transplantation

**Fig 6:**
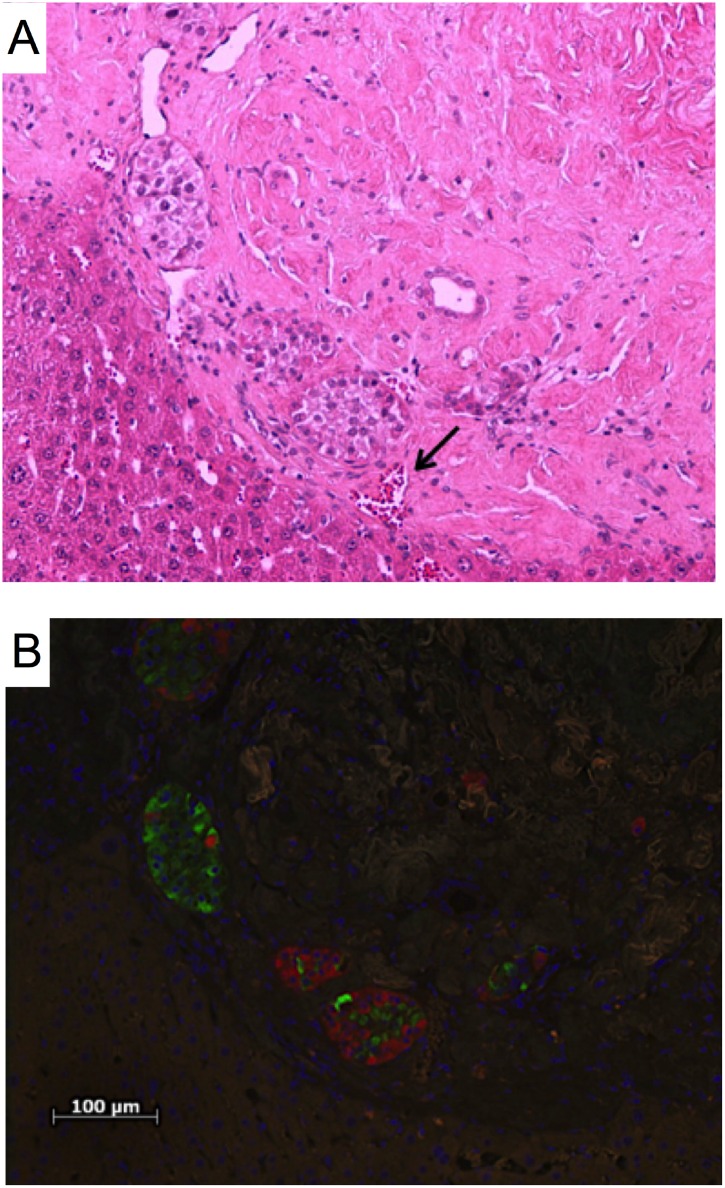
Histological analysis of explanted islet grafts 90 days post-transplantation. (A) Hematoxylin and eosin staining of a cross-section of an explanted EMP graft on the surface of the liver showing multiple islets with an erythrocyte filled blood vessels (arrow). (B) Immunohistochemistry staining of the same section confirming the presence of insulin (red) and glucagon (green).

Immunohistochemistry staining was performed 90 days post-transplantation to evaluate islet insulin and glucagon content. Human islets maintained normal morphology and size with numerous blood vessels infiltrating the microscaffold (Fig 6A). Islets stained positive for insulin and glucagon (Fig 6B), which supports the observation of normal graft function and restoration of normoglycemia.

## Discussion

In the present study, we explored the utility of a 3D lung-derived microscaffold to support islet engraftment in an alternative transplantation site. We postulated that the 3D microscaffolds provide ECM components of the islet microenvironment thereby generating favorable conditions to support islet functional survival *in vivo*.[33] EMPs have been shown to secrete insulin in a glucose-regulated manner for long periods in culture, in quantities comparable to freshly isolated islets.[32] In previous reports, supplementing islets with ECM components in culture resulted in superior islet function including collagen-IV, fibronectin,[25, 38] laminin,[24, 38] and others.[22, 23] Consistent with these results, our pilot study demonstrated potential for this approach where diabetes reversal was achieved with an islet mass as low as 500 IEQ in NOD-SCID mice with early evidence of neovascularization. Restoring the ECM microenvironment appears to reduce physiological stress experienced during and after islet isolation and consequently reducing non-immune-mediated cell death.

Cell death is one of the contributing factors of the initial islet mass loss. Anoikis, a form of apoptosis induced by disruption of cell-ECM interaction which is mediated by integrins may play an important role in islet graft loss.[39–41] Loss of peri-insular basement membrane following islet isolation has also been reportedly associated with apoptosis.[17–19] A study conducted by Pinkse *et al*. demonstrated that islets cultured on collagen-IV, laminin or fibronectin had significantly higher *in vitro* survival rates compared to collagen-I after 24 hours.[19] Blocking adhesion of β1 integrin subunit to its ECM significantly increased the number of dexoynucleotidyl transferase dUTP nick end labeling (TUNEL) positive cells in a further study.[42] The protective effect of ECM molecules could therefore offer a window for islet graft vascularization and subsequent improvement of graft survival and function.

ECM components could additionally promote infiltration of host cells into the scaffold and the interaction between ECM proteins with cell surface integrins. Leukocyte and endothelial cell infiltration facilitated neovascularization in synthetic PLGA scaffolds.[43] Salvay *et al*. demonstrated that PLGA scaffolds significantly enhanced intra-islet microvascular density.[31] This finding further suggests that ECM proteins could play a key role in islet neovascularization. Consistent with these results, we observed an abundance of blood vessels infiltrating the EMPs around the islets in a connective tissue-rich background, which likely contributed to the beneficial effect observed in islet graft function. While in previous approaches, an individual ECM component is added to synthetic scaffolds, we utilized a natural source for the EMPs, rich in ECM components capable of preserving the spatial 3D relationship in an effort to provide a near natural microenvironment.[32, 33] We utilized lung tissue as a source to produce the microscaffolds rather than pancreas because of its large surface area lined by a basement membrane. In addition to providing the 3D scaffolding that supports the architecture and cell organization found in the native environment, the resulting microscaffolds were approximately 300μm in thickness, which allowed for free diffusion of gases and nutrients, thus reducing hypoxia.[32, 33] We chose decellularized human lung microscaffolds for the human islet study component as a means to test this clinically accessible and potentially approvable source of microscaffolds for future human clinical application.

The peritoneal cavity is an alternative site for islet transplantation as it offers a larger potential space compared to the liver, while avoiding liver-associated complications such as portal thrombosis or bleeding.[44] It is a readily accessible extravascular site with adequate arterial supply, and may be accessed readily with minimally invasive surgical techniques. Furthermore, the peritoneal cavity, like the pancreas, has dominant venous drainage to the portal circulation and may thus offer potential for physiologic insulin release and hepatic first pass metabolism. We further recognize that the peritoneal cavity may have more clinical relevance as compared to the subcutaneous site. Transplantation of unmodified islets into the peritoneal cavity has consistently been associated with poor engraftment and markedly impaired function in multiple previous studies, and has not worked effectively for human islet engraftment in patients.[45–47] Consistent with these previous findings, we observed severely impaired islet function when unmodified islets were implanted intraperitoneally.

In the current study, we observed significantly enhanced functional engraftment with reversal of diabetes in both murine and human islets when transplanted intraperitoneally with EMPs. One of the limitations of using the intraperitoneal site is an inability to effectively recover implanted islet grafts due to their adherent nature post procedure.[48] Application of glucose-stimulated human-specific c-peptide assays in the present study allowed us to confirm that normoglycemia resulted from successful engraftment of human islets. Indeed, we observed substantial circulating stimulated human c-peptide levels quantifiably comparable to levels observed in our human subjects receiving intraportal islet infusions resulting in insulin-independence (1.23 ± 0.15 nmol/L in mice bearing intraperitoneal EMPs vs. 1.62 ± 0.07 nmol/L in human subjects receiving islet transplants).[49] A dose-response relationship between non-fasting blood glucose levels and c-peptide levels was observed. The EMPs stained positive for insulin and glucagon 90 days post-transplantation, supporting the contribution of the islet graft to the achievement of normoglycemia. Furthermore, mice achieved normoglycemia with an islet mass as low as 500 IEQ. To our knowledge, this is the lowest intraperitoneal human islet mass that has resulted in normoglycemia in a mouse model. Of note that, rodents are resistant to human insulin and they require significantly higher doses of insulin to reverse hyperglycemia.[50] This reduction in islet mass renews interest in the peritoneal cavity for islet transplantation and potentially for future application with stem cell-derived or xenogeneic cell transplant sources.

It has been documented that immunosuppressive agents are toxic for pancreatic islet function.[51] For instance, tacrolimus, which is one of the most effective drugs to prevent rejection, has been associated with a decrease in insulin gene expression and insulin secretion as well as graft revascularization.[51, 52] In a recent report, supplementing islets with synthetic antiaging glycopeptide was shown to be cytoprotective as it resulted in improvement of islet graft survival and insulin secretion.[52] One of the new immunosuppressive approaches is targeting the lymophocytic inotropic purinergic P2X receptor (P2X7R) that has been shown to play an important role in islet allograft rejection.[53] The use of P2X7R inhibitors delayed islet allograft rejection *in vivo*, and induced hyporesponsiveness toward donor antigens.[53] The use of anti-CD3, anti-thymoglobulin, CXCR1 and CXCR2 blockers are promising new approaches that were shown to be less toxic alternatives for the currently used immunosuppressive agents.[54–57] The use of CXCR4 antagonist mobilized autologous hematopoietic stem cells and prolonged islet allograft survival in C57BL/6 mice.[58] For patients with T1DM, the presence of autoimmune response is another factor that could compromise the transplanted islet graft function.[54] In Vergani et al., the prolonged use of low dose murine anti-thymoglobulin (mATG) with CTLA4-Ig abrogated the autoimmune response, delayed allograft rejection and prolonged islet allograft survival in NOD mouse model.[59] This novel approach reversed diabetes in newly hyperglycemic NOD mice that maintained normoglycaemia for 60 days of follow up.[59] In the present study, we have used an immunocompromised mouse model to investigate the impact of EMPs on islet graft survival and function without cofounding effects from rejection and immunosuppressive agents. In future studies, it would be desirable to transplant EMPs into immunocompetent mouse model implementing novel immunosuppressive approaches to facilitate the transition to clinical transplantation.

In conclusion, we report reversal of diabetes in an immunodeficient mouse model using either subcutaneous or intraperitoneal EMP transplantation. In this approach, we modified the local islet microenvironment by providing a wide variety of ECM macromolecules to enhance graft survival and function. Sionov *et al*, has previously characterized the EMPs demonstrating significant morphological changes, while in culture, resulting in contraction and folding of the EMP, which ultimately becomes a small sphere varying from 0.7 to 1.5 mm in diameter.[32] Furthermore, this micro-organ was shown to contain endocrine components of the natural pancreas and capable of producing insulin in glucose-regulated manner consistent with our *in vivo* findings. Of especial importance is the ability of EMPs to promote vascularization, which is an essential trait for β-cells survival. Additionally, utilization of an alternative extravascular site could potentially ameliorate IBMIR and provide an efficacious means of beta cell replacement when islet intra-portal infusion is contraindicated. This approach represents a significant improvement in bioengineered scaffolding and a transition from using synthetic biomaterials to more natural sources. This transplant technique is potentially clinically applicable and easily translatable using human-grade human-derived materials.

## Supporting Information

S1 File Long-term graft function after mouse islet transplantation. (XLSX)

S2 File IPGTTs of the transplanted mouse islets six weeks post-transplantation. (XLSX)

S3 File Long-term graft function after human islet transplantation. (XLSX)

S1 Table Reference sequences of TaqMan probes obtained from the Applied Biosystems TaqMan expression system. (DOCX)
