# Supplementary material for: Lung-Derived Microscaffolds Facilitate Diabetes Reversal after Mouse and Human Intraperitoneal Islet Transplantation
Source: PLoS One. 2016 May 26;11(5):e0156053. doi: 10.1371/journal.pone.0156053 (PMC4881949; doi:10.1371/journal.pone.0156053)
Supplement: S1 Table — (DOCX) [file pone.0156053.s004.docx]

**Table 1.** Reference sequences of TaqMan probes obtained from the Applied Biosystems TaqMan expression system.

| Gene | Accession number | ABI primer RefSeq |
| --- | --- | --- |
| TBP | **NM_003194.4** | **Hs99999910_m1** |
| GAPDH | **NM_002046.4** | **Hs99999905_m1** |
| HPRT-1 | **NM_000194.2** | **Hs02800695_m1** |
| Insulin | **NM_000207.2** | **Hs02741908_m1** |
| Pdx-1 | **NM_000209.3** | **Hs00236830_m1** |
